# A Simple Low-Cost Electrocardiogram Synchronizer

**DOI:** 10.3390/s21175885

**Published:** 2021-09-01

**Authors:** Susana Amorós, Carolina Gálvez-Montón, Oriol Rodríguez-Leor, Juan Manuel O’Callaghan

**Affiliations:** 1 School of Telecommunications Engineering, Universitat Politècnica de Catalunya, 08034 Barcelona, Spain; joan.ocallaghan@upc.edu; 2 ICREC Research Program, Health Science Research Institute Germans Trias i Pujol, Can Ruti Campus, 08916 Badalona, Spain; cgalvez@igtp.cat (C.G.-M.); oriolrodriguez@gmail.com (O.R.-L.); 3 CIBERCV, Instituto de Salud Carlos III, 28029 Madrid, Spain

**Keywords:** biomedical signals, ECG, bioinstrumentation, synchronization, trigger, medical sensor development

## Abstract

Electrocardiogram (ECG) synchronization is useful to avoid the effects of cardiac motion in medical measurements, and is widely used in standard medical imaging. A number of medical equipment include embedded commercial synchronizers. However, the use of independent synchronization modules is sometimes needed when several non-integrated instruments are used, or in the development of new medical instruments and procedures. We present a simple low-cost ECG synchronizer module based on an Arduino controller board that converts the ECG signal into a transistor-transistor-logic (TTL) one, allowing real-time medical measurements triggered at specific phases of the cardiac cycle. The device and conversion algorithm developed is optimized in vitro using synthetic and human ECG signals, and tested in vivo on three swine specimens. Error rates during the in vivo testing stage remain below the 2% of the cycles in all animals and critical false positives are less than 1%, which is sufficient for most applications. Possible algorithm updates are discussed if its performance needs to be improved.

## 1. Introduction

Electrocardiogram (ECG) synchronization is sometimes required to avoid the effects of cardiac motion in medical measurements performed on the chest and abdomen [1]. This technique is widely used in standard medical imaging techniques, including magnetic resonance imaging (MRI) [2,3], intracoronary optical coherent tomography (OCT) [4], and triggered angiography non-contrast enhanced (TRANCE) [5], to prevent motion artifacts, such as shadows and blurred contours, on images.

ECG synchronization is done by means of prospective or retrospective ECG triggering (ECG-T). Prospective ECG-T performs measurements only at a certain phase of the cardiac cycle (e.g., ventricular systole) chosen to meet the application requirements [6] and triggered by the desired physiological event (e.g., R-wave). In retrospective ECG-T, measurements are continuously performed at several phases of the cardiac cycle and afterwards correlated to the ECG phase and sorted accordingly. The optimal method depends on the application to be used [7].

Prospective ECG-T requires an algorithm to detect a specific cardiac event. Given that a large number of studies aim to automatically analyze ECGs, the literature provides numerous algorithms that can detect specific events, such as the QRS complex [8,9]. Despite this, not many synchronization devices can be found [10]. Most of them are embedded in medical equipment. However, the use of independent synchronization modules is needed when several non-integrated instruments are used, or in the development of new medical instruments and procedures.

In this work, we present a simple prospective ECG-T synchronizer that generates a transistor-transistor-logic (TTL) signal to be used in any TTL-compatible instrument. The system is based on an Arduino controller board and can be adjusted to trigger at any phase of the cardiac cycle, and to compensate for possible instrumentation delays. The code developed, 3-D print files, and component list are available online.

## 2. Materials and Methods

### 2.1. Hardware Set-Up

**Figure 1:**
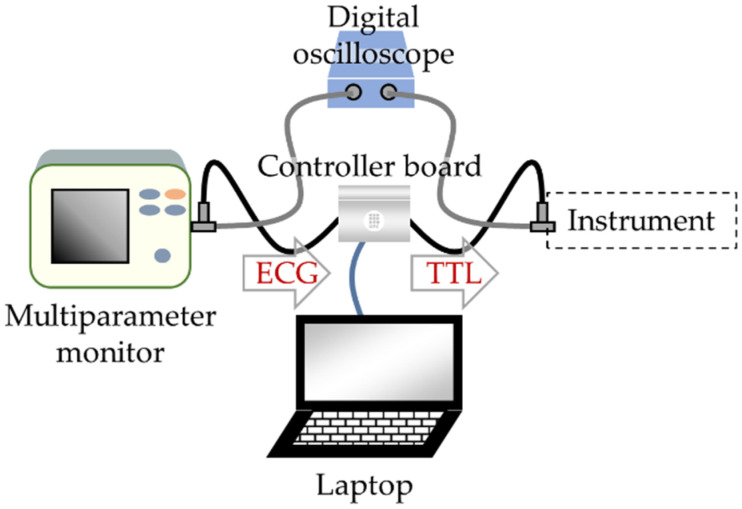
Block diagram of the hardware set-up comprising a controller board, a multiparameter monitor to capture and record the ECG, and a digital oscilloscope. The laptop and oscilloscope are used to oversee the ECG to TTL conversion during the development tests. They can be suppressed once development is complete.

As shown in Figure 1, an Arduino-based controller board (UNO R3 board, ELEGOO Shenzhen, China) was connected to a multiparameter monitor (PM-8000 Vet, Mindray, Shenzhen, China) and to the triggering port of a TTL-enabled instrument. To oversee the conversion, both connections were probed by a digital oscilloscope (PicoScope 2000 series, Pico Technology, Saint Neots, UK) and the whole system was monitored by a laptop (GL63 8RD, MSI, Taipei, Taiwan). To secure the connections, the controller board was kept in a 3-D-printed shell equipped with SMA connectors that were cabled to the input and output board pins. Coaxial cables were used to connect the various units. BNC connectors were used everywhere except at the controller ports. A pair of BNC tees were included to connect the digital oscilloscope to monitor the controller’s input and output signal.

### 2.2. Algorithm for ECG to TTL Conversion

**Figure 2:**
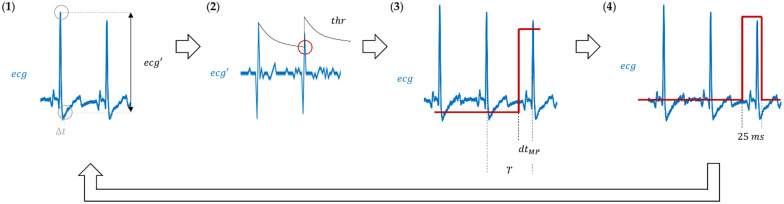
ECG to TTL conversion algorithm: (**1**) compute the first derivative, ecg′, as the difference between two samples distanced Δt; (**2**) compare ecg′ to a predefined threshold, thr (black line); whenever ecg′>thr (red circle), an R-wave is detected; (**3**) wait $T-dt_{MP}$ seconds and set a high TTL state (red line); (**4**) after 25 ms, set the TTL to low and return to step 1.

The controller board used the analog ECG signal, ecg, from the monitor as an input and the algorithm performed its conversion to a digital TTL signal as schematized in Figure 2. We used the R-wave to compute the heart rate and establish the ECG phase. To avoid false triggers from other ECG waves, and the effect of the baseline wander, a well-known low-frequency artifact appearing in ECG signals, we combined the use of a two-point finite difference estimate of the ECG derivative and a time-dependent threshold following a quasi-exponential decay [11].

**Figure 3:**
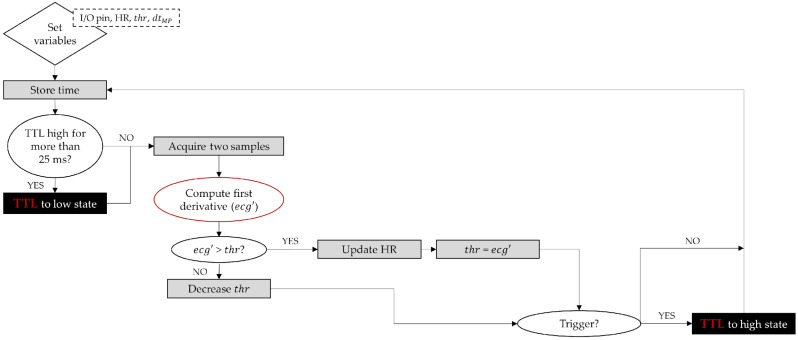
ECG to TTL algorithm diagram.

The initial phase of the ECG was established at the point where the ECG derivative is maximum (Figure 2 and Figure 3). At this point, the value of the threshold is set to be equal to the computed estimate of the ECG derivative. At 5-ms intervals, the estimate of the derivative of the ECG is re-computed and compared to the running value of the threshold. If this estimate is below the threshold, the threshold is decreased following a quasi-exponential law; otherwise, the heart rate estimate is updated comparing the current time with the previous time when the threshold was exceeded and the threshold is re-set to the current estimate of the ECG derivative. The trigger condition (Figure 3) is decided upon detection of the proper phase of the ECG cycle. In our algorithm, this is quantified through a parameter ($dt_{MP}$) that sets the time between the trigger event and the next prospective maximum of the ECG derivative. This parameter can also be used to take into account instrumentation delays, such as the time between the TTL pulse at the instrument input and the time the measurement is really performed, or delays caused by the ECG monitor equipment. Finally, our algorithm in Figure 3 is set to perform the synchronization on the raising edge of the TTL pulse, so the TTL level has to be lowered shortly after the trigger event (relative to the ECG period). Modification to trigger on the trailing TTL pulse edge is straightforward: it only requires exchanging “high” and “low” in the diagram in Figure 3.

Overall, the algorithm follows the steps below, which are illustrated in Figure 2:

1. Estimate the first derivative of the ECG (ecg′) using finite differences between two samples 5 ms apart (Δt=5 ms):
  (1) $$ecg^{\prime }=ecg\left(t+\Delta t\right)-ecg\left(t\right)$$

2. Compare ecg′ to the predefined threshold, thr.
  a. If ecg′>thr and the last R-wave detection was more than 20 ms ago, the i-th R-wave was detected. Thus, update the HR and the thr values:
    (2) $$HR\left(\frac{beats}{min}\right)=\frac{60}{T_{j}}$$
    (3) thr=ecg′
    where $T_{j}\left(s\right)=t_{i}-t_{i-1}$ is the heart rate period and $t_{i}$, $t_{i-1}$ are the times (in seconds) for two consecutive R-wave detections.
  b. Otherwise, decrease thr and return to step 1:
    (4) thr=thr·(1−x)
    where 0<x<1 Notice that x sets the decay rate of the time-dependent threshold thr. A value of x too close to 1 sets a very rapid decay of the threshold and makes the triggering vulnerable to noise and other waves in the ECG that may generate false-positive triggers. Conversely, a value of x too close to 0 may cause missing the subsequent R-wave, particularly in the presence of baseline wander.

3. Wait until $t-t_{i}>\left(T_{j}-dt_{MP}\right)$ and set a high TTL state, where t is the current time and $dt_{MP}$ is the parameter set to compensate for instrument delay and to set the ECG trigger phase.

4. Set a low TTL state if it has been high for 25 ms or more. Then, return to step 1.

### 2.3. System Adjustment and Tests

Our algorithm was optimized using the demonstration ECG signal provided by the multiparameter (D001-D002) and that from a human patient (P101-P112). In order to stress the conditions, a sinusoidal wave was added to the demonstration signal to simulate a baseline wander; likewise, the human patient was asked to make abrupt movements during tests. The main aim during the adjustment stage was to ensure that the TTL signal would only be high when an R-wave occurred. In case of a false positive (FP), i.e., a transition to a high TTL state not produced by an R-wave, the device would trigger a measurement in a phase of the cardiac cycle different to that expected. In case of a false negative (FN), i.e., a non-detected R-wave, the high TTL state immediately after an omitted R-wave might be compromised given that the computed value of $T_{j}$ might be corrupted. In the initial adjustment, performed in an electronics lab, the parameter x was set to x = 0.14% to make the trigger robust to baseline wander, to other waves in the ECG, and to noise.

Finally, our device was used in a veterinary surgical room on six anesthetized swine specimens during 29 days of follow-up. Only three swine ECGs recorded are included in this paper. They are representative of the device operation throughout the whole campaign. In some of the three swine specimens, several ECG signals were traced, recording a total of seven ECG-TTL signals (S101, S201–S204, S301–S302, where the first two characters indicate the specimen).

Animal studies were performed under the local Animal Experimentation Unit Ethical Committee and Government Authorities (Generalitat de Catalunya; Code:10388) approval, and comply with guidelines concerning the use of animals in research and teaching as defined by the Guide for the Care and Use of Laboratory Animals [12]. Three crossbreed Landrace X Large White male pigs (34.3 ± 4.3 kg) were pre-medicated with an intramuscular (IM) injection of atropine (0.04 mg/kg; BBraun, Barcelona, Spain) and sedated with dexmedetomidine (0.03 mg/kg, IM; Dexdor^®^, Orion Pharma, Espoo, Finland), midazolam (0.3 mg/kg, IM; Laboratorios Normon, Barcelona, Spain), and butorphanol (0.3 mg/kg, IM; Butomidor^®^, Richter Pharma AG, Wels, Austria). Then, anaesthetic induction was carried out with an intravenous (IV) bolus of propofol (2–4 mg/kg; Propovet^®^, Zoetis, Barcelona, Spain). Animals underwent endotracheal intubation, and anesthesia was maintained by 2% isoflurane (IsoVet^®^, BBraun), inhalation. For intra-operative analgesia, several bolus of fentanyl (0.075 mg/kg/45 min, IV; Fentadon^®^, Dechra, Bladel, The Netherlands) were delivered. At the end of the surgical intervention, Tulatromicin (2.5 mg/kg, IM; Draxxin^®^, Pfizer Animal Health, Madrid, Spain) was administered as antibiotherapy, and a transdermal fentanyl patch was applied to allow analgesic post-operative care (Durogesic^®^, Janssen-Cilag, Madrid, Spain). Finally, pigs were recovered, and housed until the experimental endpoint.

### 2.4. Availability and Cost

Once development is complete, the system can operate without the laptop and digital oscilloscope, which were only required to oversee the proper system operation in the development stage. As shown in Figure 1, the system can be placed in a single unit, inserted between the ECG monitor and the instrument to be synchronized. All parts are available at general-purpose vending sites for less than 70 €. These include Arduino UNO Rev3 or ELEGOO UNO R3, BNC male cables (×2), jumper wire cables, BNC female connectors (×2), and a 9 V 1000 mA power source (see the detailed component list including prices and vendor website links in the Data Availability Statement)

The controller board shell was 3-D-printed using a standard 3-D printer (Sigma R19 3D, BCN3D Technologies, Inc.), but it could also have been sent to an external 3-D-printing service (e.g., CIM UPC) without altering the price significantly.

## 3. Results

### Algorithm and System Operation

**Figure 4:**
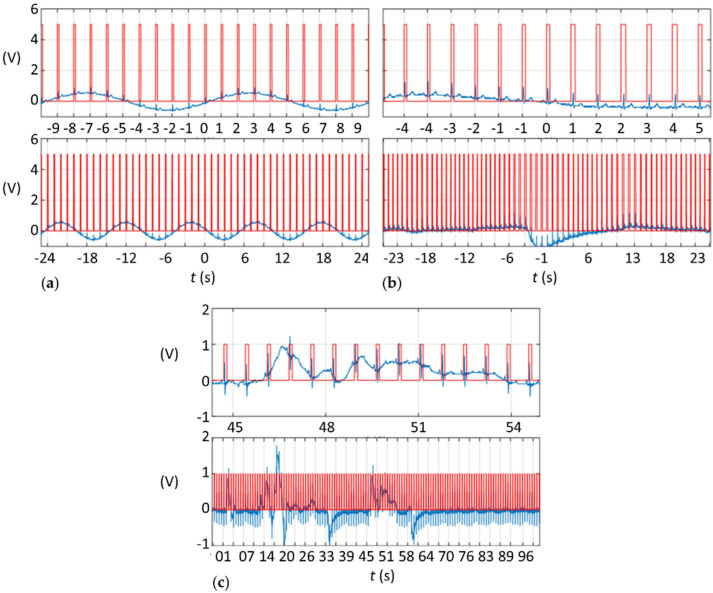
ECG (blue) and TTL (red) signals (**a**) when adding a sinusoidal wave to the demonstration ECG signal, (**b**) when using the ECG from a moving human patient, and (**c**) when using the ECG from an anesthetized swine specimen.

Overall, as summoned in Figure 4, both in vitro with the demonstration ECG signal and in vivo with human or swine subjects, R-waves were successfully detected and properly transformed to a TTL signal. Even in vivo with swine specimens and very noisy signals the conversion was performed adequately (Figure 4c).

**Figure 5:**
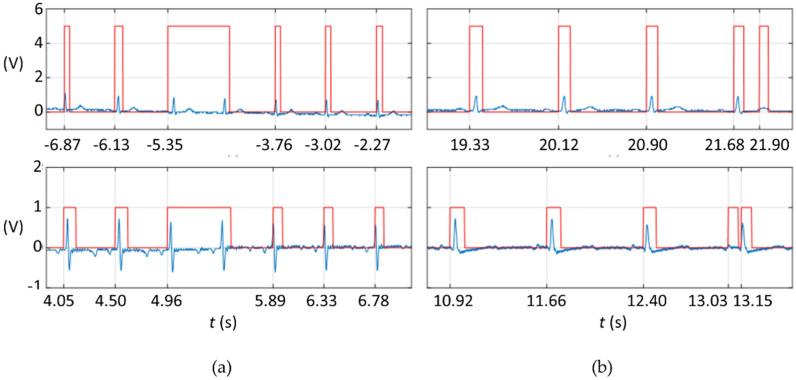
ECG (blue) and TTL (red) signals showing (**a**) false negatives when using the ECG from a moving human patient (top) and from an anesthetized swine specimen (bottom). (**b**) False positives using human (top) and swine (bottom) specimens.

Nonetheless, as detailed in Figure 5a, in vivo with the human patient and the swine specimens, some TTL high states did not become low before the next R-wave, thus omitting it, and resulting in an unexpected type of FN. Nevertheless, the next high TTL immediately after this type of FN did not appear corrupted. Some FP did occur too (Figure 5b). Table 1 quantifies the number of errors that occurred for each trace measured. Traces are chronologically sorted according to when they were measured; note that the error is reduced to zero during the adjustment stage in which the value of x was adjusted and minor code modifications were made.

**Figure 6:**
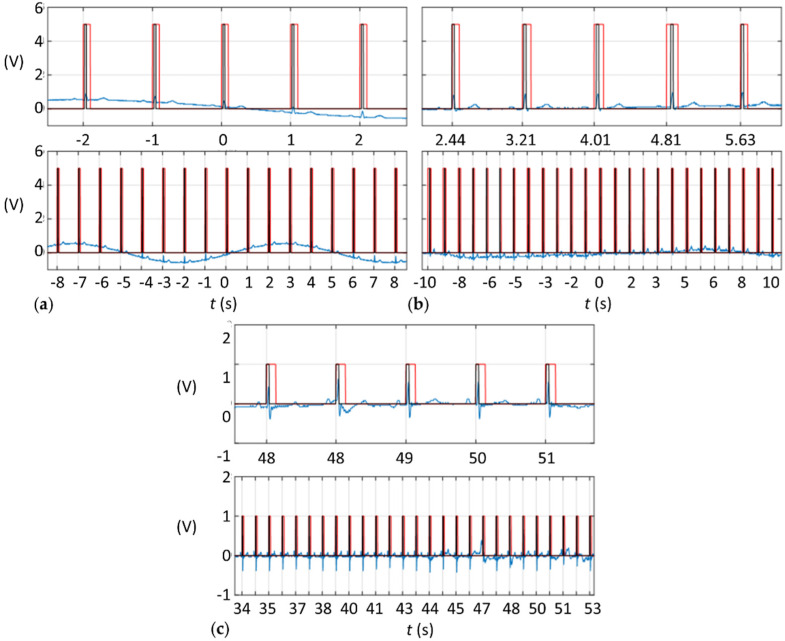
ECG (blue), TTL (red), and ideal TTL (black) signals (**a**) when adding a sinusoidal wave to the demonstration ECG signal; (**b**) when using the ECG from a moving human patient; and (**c**) when using the ECG from an anesthetized swine specimen. Note that comparisons on the TTL signals should be made on the raised edge of the pulses.

To validate the behavior of the device, the obtained TTL signal is compared in Figure 6 to the ideal TTL signal that should result from the algorithm, which can only be computed ex post using the pre-recorded ECG signal. By comparing the times when the algorithm pulls high TTL states to when they should have been pulled, the precision of the device can be estimated (Table 2). Note that Table 2 focuses only on the non-error waves, therefore excluding the FP and FN waves. On average, the TTL signal during adjustment stages was advanced relative to its proper timing by 47.04±4.62 ms with the demonstration ECG and 42.30±72.52 ms with the human patient ECG. Note that with the human ECG during the adjustment stage (P101–P107), the average was 34.94±99.37 ms, which changed to 52.60±34.92 ms during the remaining measurements (P108–P112). During testing, the TTL signal was also slightly advanced 33.27±10.83 ms (4.92±1.66% average relative to the ECG period). Note that the QRS interval of a normal ECG should not exceed 100 ms [13].

## 4. Discussion

We present a simple, cost-efficient, and versatile device that allows the synchronization of electronic measuring instruments to electrocardiographic signals with low error rates. The device was improved in vitro using synthetic ECG signals and human ECGs, and tested in vivo on six swine specimens during 29 days of follow-up.

Overall, errors during in vivo testing remain below 2% and critical FPs appear in less than 1% of the cycles. The TTL signal is slightly advanced 33.27±10.83 ms. Note that, on average, the anesthetized swine ECG signals show a periodicity of 0.66 s per cycle; hence, the resulting TTL signal is advanced by about 5±2% per cycle.

Our device has proven to be a low-budget straightforward solution able to track the ECG and provide a synchronous TTL signal that allows prospective ECG-T of any TTL-compatible electronic measuring device, preventing cardiac motion artifacts when acquiring medical measurements, and without significantly increasing the cost of the measuring system. Given the unavailability of such independent modules in the literature, the hardware and algorithms presented in this work will be useful when non-integrated instruments have to be used, or when developing new medical instruments and procedures. Nonetheless, the performance of our module was only tested on healthy specimens; its performance under several cardiopathies will be assessed using ECG signals as these become available. Updated performance information will be posted online (see the Data Availability Statement below).

The precision obtained during this study was sufficient for the application it was used for. However, in order to improve its performance, two updates may be included: (1) the analog to digital conversion of the ECG signal should be performed at the microcontroller board sample rate (in our case, 9600 bauds). Additionally, (2) the threshold value could be further decreased if the amount of FPs is to be reduced since the value used does not seem to condition the appearance of FNs. Finally, FNs may also be reduced by decreasing the threshold with a true exponential function, $thr=thr\cdot e^{-thr\cdot x}$ rather than the first-order approximation used herein.

Moreover, during this study, the device was used to trigger measurements in the event of an R-wave, but it can be customized to trigger at any phase of the cardiac cycle by simply modifying the delay between the R-wave detected and the start of the TTL pulse.

## Figures and Tables

**Table 1:** Quantification of the errors that occurred for every trace measured (# stands for “number”).

Stage	Source	Trace	# Cycles	# FP/# R-Waves (%)	# FN/# R-Waves (%)	Error (%)
Adjustment	Demo	D001	20	0.00	0.00	0.00
		D002	50	0.00	0.00	0.00
	Human	P101	64	0.00	9.38	9.38
		P102	60	0.00	6.67	6.67
		P103	61	0.00	8.20	8.20
		P104	59	1.69	10.17	11.86
		P105	67	1.49	17.91	19.40
		P106	65	0.00	1.54	1.54
		P107	64	4.69	0.00	4.69
		P108	63	0.00	0.00	0.00
		P109	66	0.00	0.00	0.00
		P110	13	0.00	0.00	0.00
		P111	64	0.00	0.00	0.00
		P112	67	0.00	0.00	0.00
Testing	Swine	S101	136	0.74	0.00	0.74
		S201	138	0.72	0.00	0.72
		S202	137	0.73	0.00	0.73
		S203	134	0.00	0.00	0.00
		S204	136	0.00	0.00	0.00
		S301	221	0.00	1.81	1.81
		S302	159	0.00	0.63	0.63

**Table 2:** Comparison between the time when high TTL states are pulled by the algorithm ($t_{a}$) and by the ideal signal ($t_{i}$): $\Delta t=t_{i}-t_{a}$ (ms) for each R-wave. FP and FN were excluded.

Stage	Source	Trace	Mean (Δt)	STD (Δt)
Adjustment	Demo	D001	49.30	5.64
		D002	44.78	3.60
	Human	P101	30.92	108.08
		P102	18.61	15.26
		P103	35.34	66.13
		P104	36.01	156.14
		P105	22.43	278.18
		P106	53.00	42.34
		P107	48.26	29.47
		P108	49.53	29.27
		P109	57.62	31.38
		P110	51.70	27.87
		P111	43.85	34.65
		P112	60.28	51.43
Testing	Swine	S101	41.07	16.60
		S201	37.12	18.68
		S202	34.54	13.59
		S203	30.45	3.74
		S204	36.62	3.41
		S301	27.01	13.47
		S302	26.06	6.36

## Data Availability

Data is contained within the article, the rest are available online at https://github.com/susanaamoros/ECGsynchronizerhttps://github.com/susanaamoros/ECGsynchronizer: Arduino code, 3D print files for the controller board case and connection diagrams. Board assembly pictures, connection diagrams, component list (including prices and vendor website links), and a dynamic update of Table 1 with extended data sets will be found online.
